# Swedish smokeless tobacco and its impact on oral health: a systematic review

**DOI:** 10.2340/aos.v85.45421

**Published:** 2026-02-06

**Authors:** Maria Bankvall, Mats Jontell

**Affiliations:** a Department of Odontology and Oral Sciences, School of Health and Welfare, University of Jönköping, Sweden; b Department of Oral Medicine and Pathology, Institute of Odontology, Sahlgrenska Academy University of Gothenburg, Sweden

**Keywords:** Health, Nicotine, Snus, Tobacco

## Abstract

**Objective:**

Swedish smokeless tobacco, or ‘snus’, has a long history of use and has undergone significant transformations, including the introduction of portion-packed snus and tobacco-free nicotine pouches. This systematic review evaluates the impact of Swedish snus on oral health, focusing on gingivitis, gingival recession, periodontitis, caries, tooth wear, and oral cleft malformations.

**Material and methods:**

The databases PubMed, Scopus, and EMBASE were used, terminating the searches on 11th June 2025. Original scientific articles written in the English or Scandinavian languages were screened by two independent researchers, finally including 26 out of 2,176 articles. The included articles were exported to the Elicit Pro library for quality assessment performed using the Joanna Briggs Institute’s Critical Appraisal Tool for Systematic Reviews. Premalignant and malignant changes were excluded from the search.

**Results and conclusion:**

The findings indicate that Swedish snus is associated with gingival recession, particularly among users of loose snus, with mechanical pressure and cytotoxic effects contributing to these lesions. Gingivitis was more prevalent among snus users, even after controlling for plaque levels, though no significant association with periodontitis was observed. Evidence regarding caries risk was inconsistent, with some studies reporting higher caries indices values among snus users, while others found no correlation. Additionally, maternal use during pregnancy was linked to a 48% increased risk of oral cleft malformations in offspring. Tooth wear and self-reported temporomandibular disorder (TMD) symptoms were also associated with snus use. These findings underscore the importance of public health measures to regulate snus use, particularly among populations such as pregnant women and adolescents, where they have increased in popularity.

## Background

Swedish smokeless tobacco, called ‘snus’, has a long-standing tradition, with documented use dating back to the 17th century [1]. The product has undergone significant transformations, evolving from an aristocratic luxury item in the 18th century to becoming a widely used tobacco product in Sweden but also abroad. A pivotal development occurred in the 1970s with the introduction of portion-packed snus, which enhanced the product’s accessibility and popularity. The snus market has experienced substantial changes in recent years, particularly through the introduction of new product categories. The traditional division between loose snus and portion snus has been complemented by tobacco-free nicotine pouches (so-called white snus) and products containing synthetic nicotine.

The development of different types of smokeless tobacco poses a challenge when it comes to studying its impact on oral health. It is complicated by factors such as brand, dose, exposure time, personal dental care, socio-economic status etc, where the latter has a particularly strong influence on oral diseases. Therefore, it is important to avoid generalizing smokeless tobacco and its impact on the oral cavity [2]. While smokeless tobacco is one of the most common causes of oral cancer in South Asia, it is uncommon for malignant changes to be caused by Swedish snus [3]. Even in the Western world, the use of smokeless tobacco differs. In the United States, moist snuff is used, which is fermented, possibly increasing the content of carcinogenic nitrosamines [4], as opposed to Swedish snus, which is pasteurized to reduce harmful bacteria and nitrosamines [5].

Except for Sweden, Swedish snus has been banned from the market in countries of the European Union since it has major health risks, but to what degree and to what extent has been extensively debated. The use of snus may impact the cardiovascular system by increasing blood pressure and impairing the vasodilatory function of blood vessels, but a direct association between snus use and the incidence of myocardial infarction or stroke has not been definitively established [2]. In addition, evidence suggests that individuals who use snus may be at an increased risk of developing type 2 diabetes compared to non-users [6]. In a recent comprehensive analysis on the use of Swedish snus and mortality, male snus users were found to have statistically significant increased mortality from all causes, cardiovascular diseases, and other causes [7]. However, the results have been questioned on methodological grounds [8]. In recent years major changes have emerged in the use of smokeless tobacco in both Scandinavia and the US with the introduction of oral tobacco-free nicotine pouches, which come in many flavors and are quickly gaining popularity and raising new public health concerns [2].

A number of systematic reviews have been published over the years, but since Kallischnigg et al., [9], no review has been published that specifically addresses snus and its effects on oral tissues. Kallischnigg et al. [9] provided suggestive evidence of an association of snus use with gingival recession and attachment loss, and of chewing tobacco with dental caries. Since then, several original articles have been published, which open avenues for new systematic reviews.

The present systematic review specifically addresses the impact of Swedish snus on gingivitis, gingival recession, periodontitis, caries, tooth wear, and oral cleft malformations. Oral premalignant and malignant diseases were not included, as the authors aim to return with a systematic review regarding these subject areas.

## Methods

The protocol of this systematic review adheres to the recommendations by the PRISMA 2020 guidelines. It was registered on PROSPERO International Prospective Register of Systematic Reviews (CRD420251068717).

### Eligibility criteria

This systematic review included original scientific articles that specifically studied the association between Swedish snus and gingivitis, gingival recession, periodontitis, caries, tooth wear, and oral cleft malformations. A defined question was created, and the following framework was used:

- Country: Country of origin of the study population
- Study design: Cohort studies and cross-sectional studies
- Population: Users of different types of Swedish smokeless tobacco in Europe with special reference to users in Scandinavia, where none of the participants used other types of tobacco.
- Comparison: Non-users of smokeless tobacco. Other types of smokeless tobacco were primarily investigated.
- Exposure: Special attention was given to the type of smokeless tobacco, although most articles did not discriminate between loose and portion-bag packed alternatives, but over time loose snus has been surpassed by portion-packed snus, which was considered in the present study.
- Primary outcome measures: Assessments related to gingivitis, gingival recession, periodontitis, caries, tooth wear, and oral cleft malformations.
- Outcome and statistics: Key findings and statistical analyses.

Articles that dealt with smokeless tobacco used in the Middle East, South Asia, and Africa were excluded. Only articles written in English or Scandinavian languages were screened. Case reports, case series, animal studies, *in vitro* research, review articles, books, single abstracts, posters, article retractions, unavailable full-text studies, letters, comments, and editorials were not included as part of this review. No US studies were available that address the adverse effects of Swedish snus, presumably because there is limited market penetration of this type of smokeless tobacco in the US.

### Databases and search strategies

Searches were made using MeSH, keywords, and free-text terms in the title/abstract, adapted to fit each database separately. The searc strategy was compiled from general and specific search terms based on clinical experience as well as results from test searches. The databases PubMed, Scopus, and EMBASE were searched using the following query: (stomatognathic disease[Mesh] OR caries[tiab] OR cari*[tiab] OR gingiv*[tiab] OR saliva[tiab] OR periodont*[tiab] OR pain[tiab] OR taste[tiab] OR cleft[tiab] AND (snus[tiab] OR snus-like[tiab] OR snuff[tiab] OR nicotine pouches[tiab] OR smokeless tobacco[tiab] OR “Tobacco, Smokeless”[Mesh])) NOT (India[Title] OR Pakistan[Title] OR Nepal[Title] OR Saudi[Title] OR Sudan[Title] OR Middle East[Title] OR Asia[Title] OR Africa[Title] OR vitro[Title] OR cessation[Title]). When using citation searching, no further articles were retrieved. The overall search strategy was developed together with librarians at the University of Gothenburg’s Biomedical Library to maximize the sensitivity. The last search was terminated on the 11th June, 2025.

### Selection process

**Figure 1:**
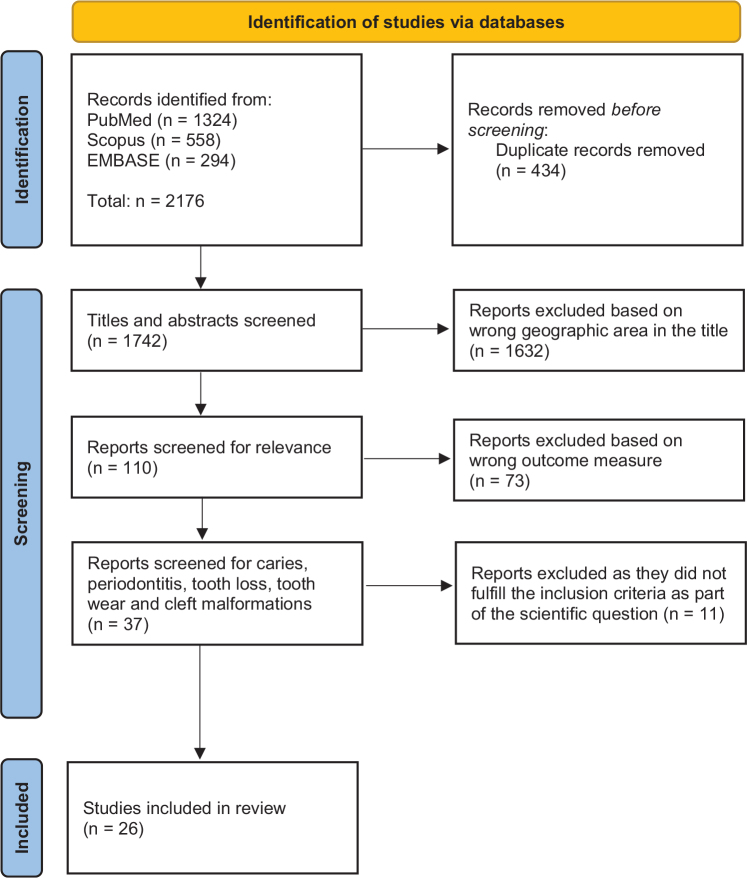
PRISMA study flow diagram.

All eligible articles were exported to EndNote (version 21) to delete duplicates. All remaining articles were then exported to Rayyan [10] for screening independently at title and abstract level by two researchers (MB and MJ) against the inclusion criteria defined for the review. Thereafter, the decisions were de-blinded and cross-referenced. Conflicting decisions were discussed until consensus was reached. Where required, the full text was retrieved and exported back to EndNote as an attached PDF document and read before a decision was reached. Full-text studies that did not meet the inclusion criteria were excluded, and the reasons for exclusion were noted. The search results are presented in the PRISMA flow diagram (Figure 1). The final included PDF documents were exported to the Elicit Pro library [11]. Elicit Pro was used to tabulate the items in the framework mentioned above. These entries were manually checked in the separate PDFs.

### Quality assessment

The quality assessments of the reviewed cross-sectional studies were performed using the Joanna Briggs Institute’s Critical Appraisal Tool for Systematic Reviews [12]. The tool uses eight critical questions related to criteria for inclusion, study subjects, exposure, measurement, confounding factors, outcomes, and statistical analysis. The three cohort studies were assessed by adding three questions to the cross-sectional tool that pertain to the follow-ups in the cohort studies. Two researchers (MB and MJ) independently assessed all included articles. The decisions were then cross-checked, and conflicting assessments were discussed and reviewed. Appraisal scores were calculated as the number of quality points received out of possible points for each respective study type, expressed as a percentage. Quality decisions and appraisal scores are presented in Table 1. Due to the limited material available for this topic, all studies were included regardless of quality, which ultimately affects the quality of the present review. Elicit Pro [11] was used to identify the specific sections in the PDFs that contained the answers to the critical questions.

**Table 1:** Quality assessment of analytical cross-sectional studies.

	Q1	Q2	Q3	Q4	Q5	Q3	Q7	Q8	
Andersson and Axel l, 1989	Y	Y	Y	Y	Y	Y	Y	Y	100%
Andersson and Warfvinge, 2003	Y	Y	Y	Y	Y	Y	Y	Y	100%
Bergst rom et al., 2006	Y	Y	N	Y	Y	Y	Y	Y	88%
Ekfeldt et al., 1990	Y	Y	N	Y	N	N	Y	Y	63%
Gunnerbeck et al., 2014	Y	Y	N	Y	Y	Y	Y	Y	88%
Hellqvist, et al., 2012	Y	Y	Y	Y	Y	Y	Y	Y	100%
Hellqvist et al., 2015	Y	Y	N	Y	Y	Y	Y	Y	88%
Hirsch et al., 1991	Y	Y	N	Y	N	N	Y	Y	63%
Hugoson et al., 2012	Y	Y	N	Y	Y	Y	Y	Y	88%
Hugoson and Rolandsson, 2011	Y	Y	N	Y	Y	Y	Y	Y	88%
Huttunen, et al., 2023	Y	Y	N	Y	Y	Y	Y	Y	88%
Jacobsen, et al., 2016	Y	Y	N	Y	Y	Y	Y	Y	88%
Kallestal and Uhlind, 1992	Y	Y	N	Y	Y	Y	Y	Y	88%
Kopperud et al., 2023	Y	Y	Y	Y	Y	Y	Y	Y	100%
Methuen, et al., 2021	Y	Y	N	N	Y	Y	Y	Y	75%
Miettinen et al., 2017	Y	Y	N	N	Y	Y	N	Y	63%
Modeer et al., 1980	Y	N	N	Y	Y	Y	Y	Y	75%
Monten et al., 2006	Y	Y	N	Y	Y	Y	Y	Y	88%
Nemeth et al., 2024	Y	Y	Y	Y	Y	N	Y	Y	88%
Rakkila et al., 2017	Y	Y	Y	Y	Y	Y	N	Y	88%
Petersson, et al., 2016	Y	Y	N	Y	Y	N	Y	Y	75%
Rolandsson et al., 2005	Y	Y	N	Y	Y	Y	Y	Y	88%
Tanner et al., 2014	Y	Y	N	Y	Y	Y	Y	Y	88%
Trullenque-Eriksson et al., 2023	Y	Y	N	Y	Y	Y	Y	Y	88%
Wickholm et al., 2004	Y	Y	N	Y	Y	Y	Y	Y	88%
Woulters et al., 1993	Y	Y	N	Y	Y	Y	Y	Y	88%
	100%	96%	23%	92%	92%	85%	92%	100%	

*Y = Yes, N = No, U = Unclear. Q1. Were the criteria for inclusion in the sample clearly defined? Q2. Were the study subjects and the setting described in detail?

Q3. Was the exposure measured in a valid and reliable way? Q4. Were objective, standard criteria used for measurement of the condition?

Q5. Were confounding factors identified? Q6. Were strategies to deal with confounding factors stated? Q7. Were the outcomes measured in a valid and reliable way? Q8. Was appropriate statistical analysis used?

### Data extraction

Elicit Pro [11] assisted in the extraction of relevant data. The extracted characteristics were modified to fit all types of included studies and clarified to enable a better overview. Data extraction was performed by one researcher (MJ) and checked by another researcher (MB). The complete data extraction is presented in Table 2. Only data relevant to the study was extracted. To be able to compare the studies more clearly, the data was reported in the same way across all studies, where possible. However, there are exceptions as not all studies presented their data in the same way.

**Table 2:** Data extraction – characteristics of the included studies.

Study	Country	Study design	Population	Comparator	Exposure	Primary outcomes measure	Outcome and statistics
Andersson andAxéll, 1989	Sweden	Cross-sectionalcase-referentstudy	252 Swedish adultmales, age range 18–66 years, meanage 36.3 ± 11.2 year	Users of loose snus were compared with portion-bag snus users	Loose and portion-bag packed snus was used by 184 (73%) and 68 (27%) study objects, respectively	Gingival recessions	Gingival recessions were found in 44 (17.8%) of 247 subjects. Five users of loose snus were excluded because of full upper and lower dentures. Among users of loose snus 42 (23.5%) subjects showed gingival recessions while only 2 (2.9%) cases were found among usersof portion-bag snus RR 8.71 (*p* < 0.009).
Andersson and Warfvinge, 2003	Sweden	Comparative cohort study	20 healthyvolunteers, meanage 34 years, number of females not stated	Intra-indvidual comparison	Loose Swedish oral moistsnus	Salivary pH	Salivary pH was significantly higher (*p* < 0.001) during the day when the subjects used snus than in the morning 6–8 h after snus use. Further, it was significantly Iower with a pinch of snus in the mouth than without snus. After having switched to the snus with both lower pH and lower nicotine concentrations, they showed a significant reduction in daily nicotine intake and developed significantly less pronounced clinical and histological changes.
Bergström et al., 2006	Sweden	Cross-sectional study	84 Swedish adult males aged 26–54 year, mean age 40.1 years	Former snus users, and never-users	Loose snus or portion-bag snus (not specified)	Periodontal bone height	No association between the use of moist snus and loss of periodontal bone height. Some cases showed loss of periodontal attachment as gingival recessions.
Ekfeldt et al., 1990	Sweden	Cross-sectional study	585 randomly selectedindividuals who in 1983 reached the ages of 20, 30, 40, 50, 60, 70, or 80 years, 306 females	Non-tobacco users	Loose snus or portion-bag snus (not specified)	Tooth wear index (IA)	Excessive tooth wear as an effect of snus. Change in R2 (factor of explanation) = 0.012 (*p* < 0.01). The use of snus and the saliva buffer capacity also seemed to be of importance for increased incisal and occlusal wear and explained 2% of the variance.
Gunnerbeck et al., 2014	Sweden	Population-based cohort study	1 086 213 live born infants, recorded in the Swedish Medical Birth Register from 1999 to 2009, 527 292 females	Non-tobacco users	Loose snus or portion-bag snus (not specified)	Oral cleft malformations	Infants of mothers who continued to use snus were 1.19 [1.01–1.41]. In contrast, in infants of mothers who stopped using snus antenatal booking, the corresponding risks were notincreased 0.88 [0.73–1.05].
Hellqvist, et al., *2012*	Sweden	Crossover comparative study	10 healthy university staff and students, mean age 36 years, 3 females	Intra-indvidual comparison	Six Swedish nicotine-free and four nicotine-containing productssnus	Carbohydrate and starch content, pH changes in plaque *in vivo*	The nicotine-containing products contained traces of carbohydrates and starches. The nicotine-free products contained up to 6.5% low-molecular-weight carbohydrates and 26.0% starch. Nicotine-containing products increased the plaque pH.
Hellqvist et al.*,* 2015	Sweden	Cross-sectional study	102 Swedish adults, age range 26 to 62 years, mean age 42.3 ± 8.7, 101 age and gender matched controls, age range 42.3 ± 8.3 years, number of females not stated	Non-tobacco users	Loose snus or portion-bag snus (not specified)	DFS, buffer capacity of saliva, saliva secretion rate, estimation of the numbers of mutans streptococci and lactobacilli, visible plaque (PLI), gingivitis (GI), plaque pH	In terms of GI, snus users had significantly higher values for the whole dentition (*p* = 0.009), as well as for the upper front area (teeth 13–23; *p* = 0.003). For PLI, there was no statistically significant difference between the two groups. The risk of developing caries was estimated to be 65.4 ± 16.2% for snus users. and 64.5 ± 16.4% for non-users (NS).
Hirsch et al., 1991	Sweden	Comparative cross-sectional study	2,145 adolescents from Göteborg, middle-class population aged 14 to 19 years. Three groups: A: tobacco users/non-tobacco users (all individuals), B: smokers/non-smokers C: snus dippers/non-snus dippers. Number of females not stated.	Non-tobacco users	Loose snus or portion-bag snus (not specified)	Caries epidemiological data DMFT, DSp, DFSp and DIP	For DMFT, DFSp, and Dip all values were significantly higher (*p* < 0.001) in all groups for tobacco users, smokers, and snus dippers compared to those not using tobacco in any form.
Hugoson et al.*,* 2012	Sweden	Cross-sectionalstudy	Randomly recruited in 1983, 1993 and 2003. Each study consisted of 130 individuals in each of the 20, 30, 40, 50, 60 and 70-year age groups. Of these, 539, 543 and 509 dentate individuals attended respective year of examination, 104 snus users in total for all three years, mean age snus users 34.9 ± 14.3, 5 females.	Non-tobacco users	Loose snus or portion-bag snus (not specified)	Buffer capacity of saliva, saliva secretion rate, estimation of the numbers of mutans streptococci and lactobacilli. Measures of tooth surfaces with visible plaque (PLI) and decayed and filled tooth surfaces (DFS) were expressed as the individual percentage of the total number of tooth surfaces (PLI% and DFS%). Radiographs were also examined.	In 1983 and 1993, there were statistically significant differencesin buffer capacity between smokers and snus users (*p* = 0.004 and *p* = 0.047 respectively), but notin 2003. In 1983 and 1993, there was a statistically significantly higherDFS in non-users and smokers compared to snus users). In 2003, there was no statistically significant difference between the three groups.
Hugoson andRolandsson, 2011	Sweden	Cross-sectional study	1,591 Swedish adults aged 20 to 70 years. Three studies, 1983, 1993 and 2003, of stratified random samples aged 20, 30, 40, 50, 60 and 70 years. The number of females is stated for each group.	Non-tobacco users	Loose snus or portion-bag snus (not specified)	Number of teeth, plaque, gingival status, probing pocket depth (PPD) ≥ 4 mm, gingival recession, height of the alveolar bone level and classification by periodontal disease experience.	In 1983 an association was found between PPD > 4 mm and snus use (OR = 3.98; *p* = 020), but not in 1993 or 2003 (NS). In 2003, there were statistically significantly lower mean PLI and GI values for snus users compared with 1993 (*p* = 0.002). Gingival recessions differed significantly between 1993 and 2003 (*p* < 0.036). snus users do not seem to have a higher risk of destructive periodontal disease.
Huttunen, et al.*,* 2023	Finland	Cross-sectionalstudy	8,552 Finnish male conscripts, mean age 19.6 years	Non-tobacco users	Loose snus or portion-bag snus (not specified)	Logistic regression analysis with DT > 0 as dependent variable and snus as independent variable	DT > 0 OR 1.05 (0.92–1.19).
Jacobsen, et al.*,* 2016	Norway	Cross-sectional study	869, age 16 years, 48.3% females	Non-tobacco users	Loose snus or portion-bag snus (not specified)	DMFS. Four bitewing radiographs. DMFS-scores was dichotomized with cut-off point DMFS = 6.	DMFS differed between snus users and non-snus users Odds ratio (OR) = 1.57 (1.12–2.21)
Kallestål andUhlind, 1992	Sweden	Cross-sectionalstudy	570 Swedishadolescents aged 16 and 18 years old, number of females not stated	Referent group without buccal attachment loss	Loose Swedish oral moistsnus	Buccal attachment loss (> 1 mm) in one or more sites	No differences in the use of smokeless tobacco between the referent and the case group were detected.
Kopperud et al.*,* 2023	Norway	Cross-sectional study	1,363 Norwegian adolescents aged 18–20 years, number of females stated for each study group	None	Loose snus portion-bag snus (10.1%) or portion-bag snus (89.4%)	Gingival recession	Gingival retractions were observed in 18.4% of the participants. Gingival retraction increased with the amount of snus boxes used.
Methuen, et al.*,* 2021	Finland	Comparativecohort study	202, 15–17 years, mean age 16.5 ± 0.5 years, 48% females	Intervention group (including physical activity and dietary counselling sessions) compared to non- intervention group	Loose snus or portion-bag snus (not specified)	DT (ICDAS criteria)	The use of snus, but not smoking, was significantly associated with decreased odds for restorative treatment need due to dental caries. DT IRR 0.3 (0.1–0.9) *p* < 0.028.
Miettinen et al., 2017	Finland	Population-based cohort register study	8,678 Finnish conscripts, mean age 19.6 years, 148 females	Non-tobacco users	Loose snus or portion-bag snus (not specified)	Self-reported facial pain and symptoms of TMD were used as outcome variables	Facial pain: OR = 1.36 (1.18–1.58).
Modéer et al., 1980	Sweden	Cross-sectionalstudy	232 Swedishschoolchildrenaged 13–14years, mean age 13.5 years, 119 boys and 113 girls	Non-tobacco users	Loose snus or portion-bag snus (not specified)	Gingivitis, plaque	snus significantly correlate (*p* < 0.001) with gingival index after controlling for plaque index.
Montén et al., 2006	Sweden	Cross-sectional study	103 19-year-old male individuals	Non-tobacco users	Loose snus or portion-bag snus (not specified)	Probing pocket depth, gingival recession, gingivitis, plaque score, clinical attachment loss (CAL)	No differences between the two groups were found regarding plaque, gingivitis, or pocket probing depth. The mean CAL amountedto 0.2 and 0.1 mm in snus users and controls, respectively (*p* < 0.05). Gingival recession OR 5.099 (1.672 – 15.549).
Németh et al.*,* 2024	Hungary	Observational study with elements of cross-sectional study design	248 ice hockey players and football players. 206 male and 42 female Hungarian students age range 12–20 years.	Non-tobacco users	Preferably portion-bag snus	Marginal bleeding score in conjunction with tooth brushing	Regular snus user group (60%) reported gum bleeding when tooth brushing compared to 37% (*p* = 0.040) in the never-used group.
Päkkilä et al.*,* 2017	Finland	Cross-sectionalstudy	3,420 Finnish male conscripts, mean age 19.6 years	Non-tobacco users	Loose snus or portion-bag snus	DMFT	OR for DMFT > 1 seems to be lower for snus users but not statistically significant.
Petersson, et al.*,* 2016	Sweden	Cohort study	1,295 19-year-olds. After 3 years, 982 of the patients were reexamined, number of females not stated	Intra-indvidual comparison	Loose snus or portion-bag snus (not specified)	DFS increment (WHO criteria)	snus users vs. non-tobacco users RR = 0.8 (0.5 – 1.3). Thereby snus was found to be unrelated to caries.
Rolandsson et al., 2005	Sweden	Cross-sectional study	80 Swedish adolescent males aged 16–25 years	Non-tobacco users	Loose snus or portion-bag snus (not specified in relation to the outcomes)	Number of teeth, restoratlons, plaque and glnglvltls, pocket depth, gingival recessions	There was not a significant difference in the number of teeth, restored tooth surfaces presence of plaque, gingivitis, or pocket depth between snus users and non-users. Gingival recessions were found in 7 out of 40 snus users.
Tanner et al., 2014	Finland	Cross-sectionalstudy	8,537 conscripts, 148 females	Non-tobacco users	Loose snus or portion-bag snus (not specified)	DT and DMFT values were dichotomised as follows; DT > 0 and rest and DMFT > 1 and rest	snus users DT > 0, 1.04 (0.92 – 1.17) and DMFT > 1, 1.12 (0.98 – 1.28)
Trullenque-Eriksson et al.*,* 2023	Sweden	Retrospective registry-based cohort	345 Swedish adults examined at age 19 years, followed up to 31 years, 56.2% women	Non-tobacco users	Loose snus or portion-bag snus (not specified)	Number of teeth, plaque score, marginal bleeding score, probing pocket depth (PPD)	No significant association between snus use and onset of periodontitis (≥ 2 teeth with PPD ≥ 6 mm); OR: 1.65 (0.63–4.32), *p* = 0.308.
Wickholm et al., 2004	Sweden	Cross-sectionalstudy;Registry-basedstudy	1,674 Swedish adultsaged 31–40years, 50.8% women (mean age 36.7 ± 2.9 years) and 49.2% males (mean age 36.7 ± 2.8 years)	Non-tobacco users	Loose snus or portion-bag snus (not specified)	Pl I > 2.0, GI > 2.0, CI > 2.0, recessions PD > 5 mm	Ever snus users had a somewhat higher prevalence of gingival inflammation, gingival recessions, and teeth with PD > 5 mm than never users of tobacco. The association between snus use and periodontal disease was, however, not statistically significant, although there was an indication of an association with former snus use compared to never use.
Woulters et al.*,* 1993	Sweden	Cross-sectional study	723 Swedish adults, age range 20–80 years,378 men and 345 women	Non-tobacco users	Loose snus or portion-bag snus (not specified)	The linear aiveolar bone height (B)/root length (R).	There was no statistically significant relationship between snusing and B/R (*P* > 0.05).

## Results

### Description of included studies

In total, 2,176 articles were retrieved from the three databases PubMed, Scopus, and EMBASE. After removing duplicates, 1,742 articles remained. Out of those, 110 articles passed the title and abstract screening, of which 26 fulfilled the inclusion criteria and were eligible for inclusion after full-text screening. The data extraction and selection process are presented in the PRISMA diagram (Figure 1).

The methodological quality ranged from 23 to 100 % between studies and 63 to 100 % within single studies (Table 1). The lowest score for a single item was 23 % and was due to the omission of the types of snus that the study participants used. This means that in close to three-quarters of the studies, it was not possible to relate the outcome measure to a specific type of snus. However, as the authors assumed there was a mixture of loose and portion-packed snus in the studies where the type of snus used was not specified, the figure would be significantly higher. In most studies, the study population was clearly described, but since they often represented specific populations, generalizability is limited due to potential selection bias. However, because of the low number of studies included, no articles were excluded after quality assessment regardless of appraisal score.

The included studies were conducted between 1980 and 2025 in English. Studies were published from Finland, Hungary, Norway, and Sweden, whereas there were no studies from countries outside of Europe. There was a dominance of articles from Sweden, and surprisingly no articles were published from Denmark, which also belongs to the Scandinavian countries. The studies varied in geographical location, including schools, universities, military facilities, sports facilities, a construction site, a shipping site, and public and private practices.

Patient data was retrieved from various sources such as interviews, questionnaires, clinical examination, radiographic assessment, saliva samples, pH measurement, impressions of the upper and lower jaw, and medical records from the Swedish population-based register, the SkaPa register (Svenskt kvalitetsregister för Karies och Parodontit), the Jönköping study register, and the Physical Activity and Nutrition in Children (PANIC) study register. Sample sizes ranged from 10 to 8,552 for caries, 80 to 1,674 for periodontitis and 585 to 1,086,213 for other conditions such as tooth wear and oral cleft malformations. There was a dominance of males in the study populations, where some studies did not include females at all. The age ranged from 12 to 70 years in studies related to caries and in studies related to periodontitis. For other conditions, this was not calculated because they represented single studies. The complete data extraction with study characteristics is presented in Table 2.

### Studies related to caries

Among the studies there were contradictory results with regard to the increased risk of caries due to the use of Swedish snus even though the majority showed no correlation. This was also the case in studies of behavioral patterns.

#### Plaque and saliva

The average salivary pH was significantly higher (*p* < 0.001) during the usage of snus and increased even more shortly after snus removal [13]. Also, saliva secretion rates were found to be higher in users of snus than in non-tobacco users [14], which was however not supported in the study by Hugosson et al., when studied over time [15]. The buffering capacity was not found to be different between snus users and non-snus users or the amount of *streptococcus mutans* or *lactobacillus* [14]. For the plaque pH, nicotine-containing snus increased it as opposed to nicotine-free products, which lowered the pH [16].

#### Caries indices

There was a positive correlation for DMFT (decayed, missing, filled teeth) and the number of years that snus had been used (*p* < 0.05) [17]. Also, Jacobsen et al*.* [18] supported this observation when studying DMFS (decayed, missing, filled surfaces) and reported an odds ratio (OR) of 1.57 (1.12–2.21). Most articles, however, did not find a positive correlation between snus and caries when assessing DFS (decayed, filled surfaces) [14, 15, 19] and DT (decayed teeth) [20, 21]. This lack of correlation was also observed over time [22].

#### Behavioral patterns

The consumption of sports drinks is particularly common among those who exercise frequently, with studies showing that snus users, who tend to be more physically active, have higher consumption rates of sports drinks [21]. However, some physically active individuals and snus users demonstrate better dietary habits in other areas, such as lower intake of snacks between meals and less frequent consumption of cookies and buns [14, 23], which could help offset the cariogenic effects of sports drinks. The protective effect of exercise against caries may be partially explained by the fact that physically active individuals tend to have healthier behaviors overall, although this benefit can be compromised by the frequent consumption of carbohydrate-rich sports drinks and reduced salivary flow during prolonged physical activity [21].

### Studies related to periodontitis

The studies that were included showed that Swedish snus is associated with gingivitis and gingival recession but were not indicative of an increased risk of developing periodontitis.

#### Plaque and gingivitis

No significant difference in Plaque Index (PLI) was observed between snus users and non-users [14, 19]. However, when examining the Gingival Index (GI), snus use showed a significant positive correlation after adjusting for plaque levels (*p* < 0.001; [24]). This association was further supported by Hellqvist and coworkers [14], who reported a mean ± SD for the GI of the full dentition of 20.4 ± 18.2 in snus users versus 14.4 ± 13.9 in non-users (*p* = 0.009). In the anterior maxillary region, the corresponding values were 14.9 ± 20.6 and 7.7 ± 11.9, respectively (*p* = 0.003).

In a questionnaire-based study, 60% of current snus users reported gum bleeding, compared to 37% among never-users (*p* = 0.04; [25]). In contrast, Trullenque-Eriksson et al. [26] found no significant association between snus use and marginal bleeding scores, a finding consistent with results from the cohort study by Rolandsson et al*.* [19] and the study by Montén et al. [27].

#### Gingival recession

These were observed in only 2.9% of individuals using portion-bagged snus, compared to 23.5% among users of loose snus (*p* < 0.005) [28]. The form of the product, loose versus portioned snus, emerged as the factor associated with the highest relative risk (RR = 8.7, *p* < 0.009) for the development of caries. In a separate study, Rolandsson et al. [19] reported that 17% of snus users exhibited gingival recession, whereas none was observed among non-users. These recessions were most pronounced in the anterior region, typically corresponding to the site of snus placement. Kopperud et al. [29] similarly noted a 34% increase in the odds of gingival recession for each additional year of snus use. Supporting these findings, Montén et al. [27] reported an OR of 5.10 (95% confidence interval [CI]: 1.67–15.55), further indicating a significant association between snus use and gingival recession.

#### Pocket probing depth

The following studies assessed probing pocket depth (PPD) as an indicator of periodontal disease in relation to snus use. Rolandsson et al*.* [19] measured PPD at four sites per tooth and reported no significant differences between snus users and non-users. No periodontal pockets deeper than 4 mm were observed. Similarly, Bergström et al*.* [30] also using four measurement sites per tooth, found that pockets exceeding 3 mm were rare, with a mean PPD of 1.94 mm. No statistically significant differences were noted between current snus users, former users, and non-users. Montén et al. [27], who measured PPD at six sites per tooth, likewise, found no significant differences between groups, with mean pocket depths of 2.3 mm among snus users and 2.4 mm among controls. Hugoson & Rolandsson [31] recorded PPD ≥ 4 mm as part of their periodontal assessment and concluded that snus use was not significantly associated with increased pocket depth, in contrast to smoking, which was linked to a higher prevalence of PPD ≥ 4 mm. The most recent study, by Trullenque-Eriksson et al*.* [26], defined PPD ≥ 6 mm at two or more teeth as a clinical marker of periodontitis. No significant association between snus use and increased PPD was found.

### Studies related to oral cleft malformations, tooth wear, and TMD

#### Oral cleft malformations

One population-based cohort study including 1,086,213 infants in Sweden, where 11 461 infants had mothers using snus, showed that use during early pregnancy increased the risk of oral cleft malformation (*n* = 31) by 48 % (OR 1.48; 95% CI 1.00–2.21) [32]. In contrast, in infants of mothers who stopped using snus before the antenatal booking, the corresponding risk was not increased (OR 0.71 95% CI 0.44–1.14). Overall, high maternal age (< 35 years), a Nordic country of birth, chronic hypertension or preeclampsia in the mother, multiple births, and infant male sex were associated with increased rates of oral cleft malformations, while pre-pregnancy diabetes was reported to reduce the rate.

#### Tooth wear

Unexpectedly, the use of Swedish snus has been shown to be associated with other oral health problems as well. Snus has been associated with the increase of tooth wear based on data from 585 men and women (1:1), aged 20–80 years (*p* < 0.01; [33]). Furthermore, a reduced number of teeth, male sex, occurrence of bruxism, increasing age, and a reduced saliva buffer capacity influenced the degree of incisal and occlusal wear.

#### Temporomandibular disorders

Also, the use of snus has been associated with self-reported temporomandibular disorders (TMD) symptoms in a group of predominantly Finnish male conscripts increasing the risk for all TMD symptoms, especially for facial pain (OR 1.36 95% CI 1.18–1.58) but not jaw clicking [34].

## Discussion

The present systematic review was performed to assess the potential adverse effect of Swedish snus as a contributor to certain non-malignant oral conditions. We recognize the influence of socio-economic status, which has not been the focus of this study, and acknowledge this as important to highlight in future studies. Regarding caries, DMFT values were found to be significantly higher (*p* < 0.001) in Swedish snus users compared to non-users [17]. The authors acknowledge that confounding factors such as diet, oral hygiene, and exposure to fluoride may have contributed to the results. In the review by Kallischnigg et al*.* [9], this study was also identified, but since then no systematic review that specifically addresses Swedish snus and its potential to contribute to caries development has been published. A more recent and comprehensive study [18] using a multivariate regression analysis, indicated that use of Swedish snus showed a strong independent association with prevalence of manifest dental caries. The time difference between this study and the study by Hirsch et al. does not indicate that portion snus is less carcinogenic than loose snus as from the 1990s onward, the use of portion snus increased steadily, particularly among younger users and women. It is noteworthy that some nicotine-free products contained up to 6.5% low-molecular-weight carbohydrates and 26.0% starch, which creates the conditions for dental caries [16]. However, most articles [13–16, 19–23, 35] did not find any statistically significant correlation between Swedish snus and an increased risk of developing caries. Since there are no studies of white snus or tobacco-free snus, it is not possible to evaluate the risk of caries.

There is substantial evidence that the use of Swedish snus is associated with gingival recession [19, 27–31, 36, 37]. In the study by Andersson and Axéll [28], gingival recession associated with snus use was identified in 23.5% of the individuals among users of loose snus and in only 2.9% among users of portion-packed snus. This result is also indirectly supported by Hugoson and Rolandsson [31], who found a decreased incidence of gingival recession over time even though they did not specify the number of loose snus and portion-packed snus users, given the fact that the use of portion-packed snus was more common in 2003 compared to 1993. Montén et al. [27] reported an even higher prevalence of gingival recession, 42% among snus users compared to 17% in non-users, even though a larger proportion of users in their cohort likely consumed portion-packed snus. The discrepancy between these findings may be attributed to differences in the definition or clinical criteria of gingival recession. In a binary logistic regression model, Kopperud et al. [29] found that the odds of exhibiting gingival recession increased by 34% for each additional year of snus use. Notably, the occurrence of gingival recession appears to be independent of oral hygiene status [27, 29], supporting the hypothesis that both mechanical pressure and the cytotoxic effects of snus contribute to the development of these lesions.

Although the presence of plaque and gingivitis does not necessarily result in periodontitis, it is nonetheless important to eliminate chronic inflammatory conditions, as these may hypothetically contribute to other adverse health outcomes [38]. In the present review, users of snus exhibited significantly higher levels of gingivitis (as measured by the GI) compared to non-users, even after controlling for plaque levels (plaque index) [14, 24]. These findings are noteworthy, as they suggest that snus may independently induce gingival inflammation. In the study conducted by Modéer and colleagues in 1980, the participants were likely to have predominantly used loose snus, whereas in the 2015 study by Hellqvist et al., the opposite is presumed. However, data are conflicting as no association between Swedish snus and gingivitis has also been reported [27, 31].

Gingival recessions should not be conflated with periodontitis, as was unfortunately the case in a recently published systematic review and meta-analysis on smokeless tobacco and periodontitis [39]. Periodontitis arises from a complex interplay of risk factors, among which dental plaque plays a central role [40]. In the systematic review by Kallischnigg et al. [9] covering scientific papers from 1963 to 2007, none of the included studies on Swedish snus and periodontal disease showed any significant correlation [27, 30, 36]. Two additional studies from that time period were not indicative of periodontal disease [41, 42]. Since then, the cohort study by Trullenque‐Eriksson et al. [26], including subjects followed between 23 and 31 years, did not report any increased incidence of periodontitis, although the periodontitis was assessed solely on the presence of deep periodontal probing (PPD). In 1983, an association between PPD > 4 mm and snus use (OR = 3.98; *p* = 020) was reported by Hugosson and Rolandsson [31], but this association was not found in the analyses from 1993 or 2003.

The finding that maternal snus use during pregnancy is associated with an increased risk of oral cleft malformations in offspring is of considerable concern [32]. Compared to non-users, snus users were in the study more likely to be teenage mothers. Furthermore, in 2023, 26% of upper secondary school students in Sweden reported current use of snus, compared to 13% in 2012 – the lowest recorded level. The increase has been particularly marked among female students, with the prevalence of snus use rising from 4% to 21% over the past decade [43]. The newer tobacco-free form of snus now predominates and contains nicotine levels comparable to those found in traditional snus, the compound that Gunnerbeck and colleagues [32] identified as the most plausible teratogenic agent. These findings underscore the critical importance of enforcing existing regulations, including the minimum legal age of 18 years and the prohibition on marketing targeting individuals under the age of 25. Furthermore, considering its teratogenicity, snus should not be recommended as a safe alternative to smoked tobacco products.

This systematic review highlights the need for further research on newer tobacco-free nicotine products and their potential oral health risks since they have increased in popularity especially among women [44] and teenagers [45]. These findings underscore the importance of public health measures to regulate snus use, particularly among populations such as pregnant women and adolescents.
